# Delivering an mHealth Adherence Support Intervention for Patients With HIV: Mixed Methods Process Evaluation of the Philippines Connect for Life Study

**DOI:** 10.2196/37163

**Published:** 2022-08-12

**Authors:** Cara O'Connor, Katerina Leyritana, Aoife M Doyle, Isolde Birdthistle, James J Lewis, Randeep Gill, Edsel Maurice Salvaña

**Affiliations:** 1 Faculty of Epidemiology and Population Health London School of Hygiene and Tropical Medicine London United Kingdom; 2 Sustained Health Initiatives of the Philippines Mandaluyong Philippines; 3 Medical Research Council International Statistics & Epidemiology Group London School of Hygiene and Tropical Medicine London United Kingdom; 4 Y Lab, the Public Services Innovation Lab for Wales School of Social Sciences Cardiff University Cardiff United Kingdom; 5 Johnson & Johnson Global Public Health London United Kingdom; 6 Institute of Molecular Biology and Biotechnology National Institutes of Health University of the Philippines Manila Manila Philippines; 7 Section of Infectious Disease, Department of Medicine University of the Philippines College of Medicine University of the Philippines Manila Manila Philippines

**Keywords:** mobile health, mHealth, adherence, HIV, antiretroviral therapy, process evaluation, Philippines, men who have sex with men, MSM, mobile phone

## Abstract

**Background:**

The Philippines HIV epidemic is one of the fastest growing epidemics globally, and infections among men who have sex with men are increasing at an alarming rate. Connect for Life Philippines is a mobile health (mHealth) intervention that supports antiretroviral therapy (ART) adherence in this key population through individualized voice calls and SMS text messages.

**Objective:**

The objective of this process evaluation is to assess the intervention reach, dose delivered and received, fidelity, and acceptability and to describe contextual factors affecting the implementation of an mHealth adherence support intervention for patients on ART in a clinic in Metro Manila, Philippines.

**Methods:**

A mixed methods process evaluation approach was used in an observational cohort study. Quantitative data sources for the process evaluation were call and SMS text message logs obtained from the mHealth platform and questionnaires collected at 12-, 24-, and 48-week study visits. Qualitative data were collected from process reports and through a series of focus group discussions conducted with a subset of participants during the intervention development phase, after an initial 8-week pilot phase, and at the end of the study.

**Results:**

The 462 study participants received 31,095 interactive voice calls and 8234 SMS text messages during the study. Owing to technical issues, intervention fidelity was low, with only 22.1% (102/462) of the participants receiving reminders via voice calls and others (360/462, 77.9%) receiving only SMS text messages during the intervention. After 48 weeks in the study, 63.5% (293/462) of the participants reported that they would be quite likely or very likely to recommend the program to a friend, and 53.8% (249/462) of the participants reported that they benefited quite a bit or very much from the intervention. Participants who were on ART for <6 months at the beginning of the study and those who received the daily or weekly pill reminders were more likely to report that they benefited from the intervention (*P*=.02 and *P*=.01, respectively).

**Conclusions:**

The Connect for Life intervention had high participant satisfaction and acceptability, especially among those who received high dose of the intervention. However, poor reliability of local telecommunication networks had a large impact on the intervention’s usability, fidelity, and dose received.

## Introduction

### Background

The HIV epidemic in the Philippines is one of the fastest growing epidemics globally, with 207% increase in new HIV infections and 388% increase in AIDS deaths from 2010 to 2020. In 2020, an estimated 73% of people living with HIV in the Philippines knew their status and 44% of people living with HIV were on antiretroviral therapy (ART) [1-4]. In 2 studies of cohorts of patients with HIV in Manila, 84% to 90% of patients who started ART had achieved viral suppression [4,5]. Most new and existing HIV infections occur among men who have sex with men (MSM) [3]. Improving treatment coverage, retention, adherence, and viral suppression are key to slowing the spread of HIV in the Philippines. Unfortunately, widespread stigma, lack of knowledge, and barriers to accessing care pose a challenge to engaging patients in testing and ensuring high levels of adherence to ART and retention in care [6-8]. High rates of first-line treatment failure, loss to follow-up, and suboptimal treatment adherence lead to poor outcomes in many patients with HIV in the Philippines [9,10].

This paper describes the process evaluation of a mobile phone technology for health (mobile health [mHealth]) intervention for people living with HIV in Metro Manila, Philippines. To support ART adherence, the intervention, Connect for Life, provided patients with HIV with individualized voice calls and SMS text messages, pill reminders, appointment reminders, symptom reporting, health tips, and adherence feedback.

The Connect for Life platform was developed by Janssen Global Public Health, and before adaptation for the Philippines, its versions were piloted in India and Uganda. The mMitra (mobile friend) project in India aimed to improve maternal health outcomes through health messages to pregnant women [11,12]. The Treatment Advice using Mobile Alerts project in India [13,14] and Call for Life Uganda [15,16] supported ART adherence among people living with HIV.

### Process Evaluation of mHealth Interventions

As mHealth technologies have become widespread in low-income and middle-income countries, mobile phone interventions have become increasingly popular in the global health and development sectors as an inexpensive and efficient way to communicate and deliver services. Several trials have shown that mHealth approaches show potential for improving self-management of chronic diseases, including adherence to HIV medications [17-21], whereas systematic reviews show mixed outcomes of mHealth interventions and highlight the need for more rigorous evaluation methods and longer follow-up periods in mHealth studies [22-31].

Trials assessing mHealth adherence interventions for HIV often do not include process evaluations to examine the fidelity and quality of the intervention delivery, causal mechanisms for the health outcomes, contextual factors affecting the delivery, and costs to implement [29,32,33]. For mHealth interventions, current guidance suggests that practitioners should also include a minimum set of information about the content, context, and technical features of the intervention, including aspects such as ease of use, content quality, privacy and security, service quality, personalization, and perceived enjoyment [34-37].

Process evaluations of SMS text messages and interactive voice response systems (IVRSs) have examined fidelity, reach, dose delivered, and user satisfaction for projects ranging from water and sanitation to prevent diarrheal disease [38]; airline pilot fatigue [39]; and prevention of weight gain, smoking, or HIV among young people [40-42]. A systematic review of mHealth projects in Africa found that in projects where acceptability and usability of mHealth technology among participants was measured, it was generally high. However, infrastructure issues (unreliable network and internet and electricity access) were frequently cited as key challenges in delivery [24].

The success of mHealth projects in achieving the intended health outcomes is almost entirely dependent on the adaptation and delivery of the intervention in local contexts. Having a complete understanding of the implementation process of an mHealth intervention can enable practitioners to interpret the outcomes and replicate the intervention in other contexts. Therefore, we performed a process evaluation alongside the Connect for Life Philippines prospective cohort study. The process evaluation examined the fidelity, dose delivered and received, reach, usability, acceptability, and cost of the Connect for Life Philippines intervention.

## Methods

### Recruitment

The study was conducted at the Sustained Health Initiatives of the Philippines (SHIP) Clinic, a low-cost, private facility in Metro Manila, a city with approximately 13 million people in the predominantly Catholic country of the Philippines.

SHIP Clinic provides HIV primary care and wraparound services to approximately 900 people living with HIV. Approximately 98% of SHIP’s clients are MSM, with an average age of 30 years at initial consultation. Most are full-time or part-time employees. The clients come from all regions of Metro Manila, and some live in other provinces.

Recruitment into the Connect for Life study occurred in person at the study site between October 2016 and December 2017. As patients checked in for their routine clinic visits, the study coordinator approached all patients seated in the clinic waiting room, briefly introduced the study following a recruitment script, elicited their interest in participating, screened them for eligibility, completed the informed consent process, and provided a brief orientation to the intervention.

### Connect for Life Mobile Phone ART Adherence Support Intervention

**Figure 1:**
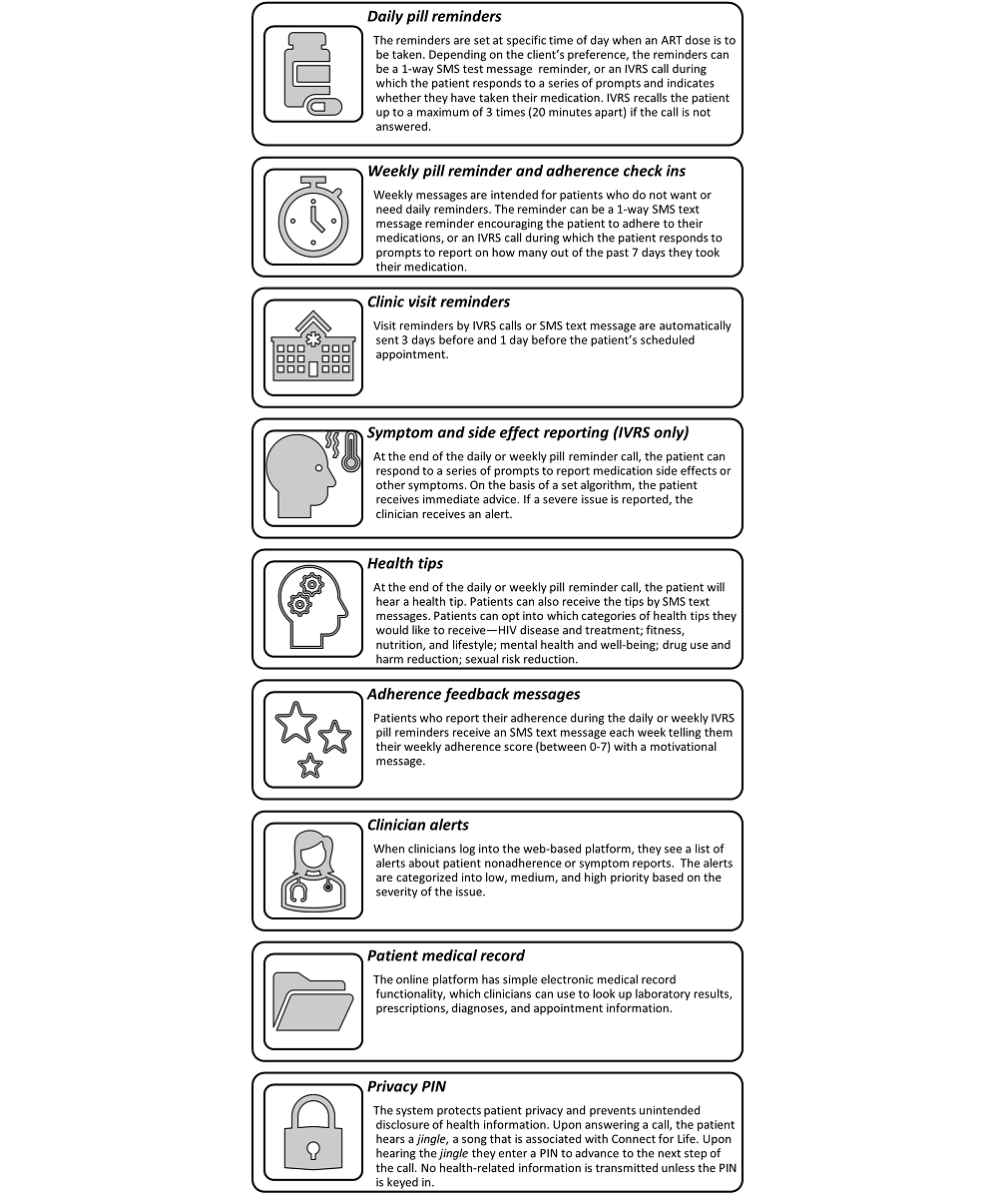
Connect for Life Philippines mobile health intervention functions. ART: antiretroviral therapy; IVRS: interactive voice response system; PIN: personal identification number.

The study team worked with IT specialists and public health professionals from Jannsen Global Public Health, University of the Philippines, and local IT companies to develop the content and functionality of the Connect for Life mHealth platform (Figure 1). Connect for Life is a technology built on the Mobile Technology for Community Health (MOTECH; Grameen Foundation) open-source software platform [43]. It enables health facilities to connect to patients via their mobile phones through IVRS call flows or SMS text messages. As Connect for Life works through phone calls and SMS text messages, it does not require the user to have a smartphone, install an app, or have mobile internet connection. This makes it accessible to a wide range of users in the Philippines, where, in 2015, mobile phone penetration was high, but smart phone coverage and internet access were low (with 113 mobile subscriptions per 100 people, 99% of the population reached by network coverage, and 22% of the population owning a smart phone) [44-46].

The study team tailored the Connect for Life platform for the Philippine context. Some existing features were retained, such as reminders sent on the recipient’s preferred days and times, health tips, and symptom screening. New features were developed, such as medical record functionality and adherence feedback scores. Clinicians at the study site developed new content for the voice and SMS text messages, which were recorded by a local voice talent agency. During the formative study and intervention development stage, a series of focus groups were conducted to engage with patients at the clinic about their adherence behaviors and preferences for configuration and content, and their feedback was incorporated to ensure that the intervention was tailored to the target population [47-49].

The Connect for Life system was installed in a secure cloud server environment and linked to a local telecom provider through application programming interface integration to execute calls and SMS text messages. A local IT service provider was contracted to monitor server functionality, install software updates, and troubleshoot technical issues. The Connect for Life software developers provided in-depth technical training and software documentation to the local IT provider and training for the clinical staff on how to use the Connect for Life web-based platform.

**Figure 2:**
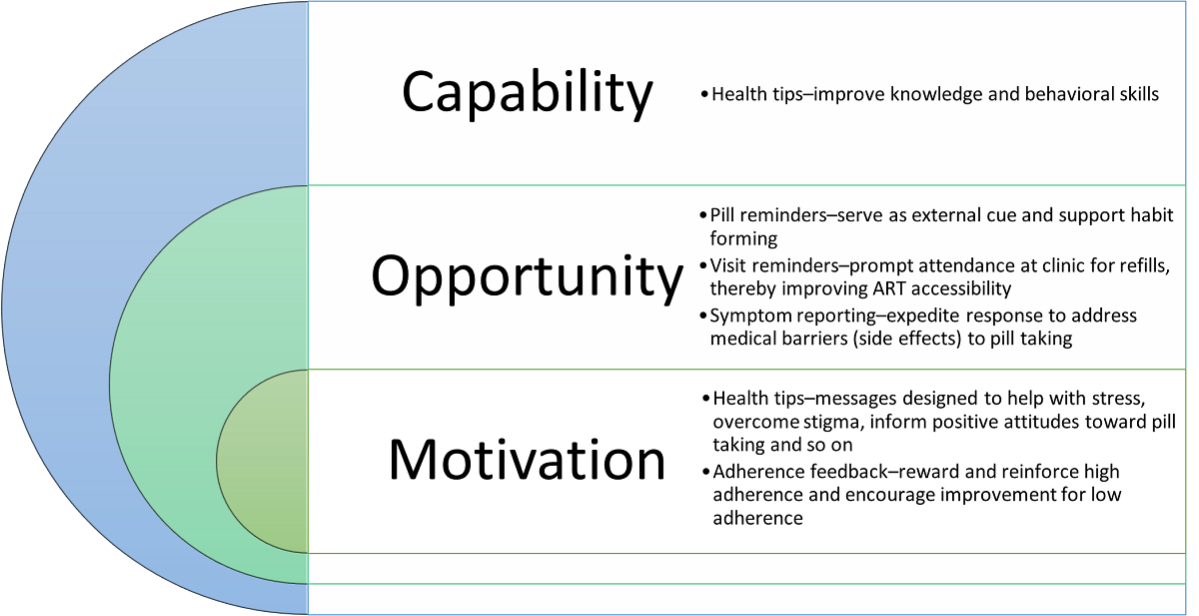
Intervention theory of change. ART: antiretroviral therapy.

The intervention development process was guided by the Behavior Change Wheel and the Capability Opportunity Motivation–Behavior model developed by Michie, Atkins, and West [50-52]. Behavior change techniques related specifically to ART adherence were informed by the information-motivation-behavioral skills model of ART adherence [53]. Each service in the intervention package was designed to address ≥1 of the 3 main components that drive behavior in the Capability Opportunity Motivation–Behavior model, as outlined in Figure 2 [47,48].

### Data Collection and Analysis

A mixed methods approach was used with qualitative data embedded in the experimental design of the 48-week prospective cohort study [54]. The design allowed us to assess participants’ use of and experience with the system and use quantitative and qualitative analyses to generate complementary data about acceptability, usability, and the impact of contextual factors on the intervention.

The process evaluation measures were based on the framework proposed by Linnan and Steckler [55], which defines the approach to adequately describe the context, reach, dose (delivered and received), acceptability, and fidelity of the intervention. Additional aspects related to reporting on mHealth technology were included based on guidance from the mHealth Evidence Reporting and Assessment checklist [36].

The process evaluation questions, tools, methods, and data sources are described in Multimedia Appendix 1.

To measure the fidelity and dose of intervention delivery, records from the mHealth platform detailing the services received by each participant were exported. To understand the usability and acceptability of the intervention, participants completed self-administered paper-based questionnaires at 3 time points during the study. Where questionnaires had blank or missing fields, all available data points were included in the analysis. Data distributions were explored to categorize the responses to the questionnaires. Associations between acceptability of the intervention and independent variables (time point, treatment experience, and reminder frequency) were calculated using chi-square tests. Data analysis was conducted using Stata 15.

Qualitative feedback was collected in several ways: routine monthly process reports from clinicians to document implementation successes and challenges, comments recorded on the acceptability questionnaires, and a series of focus group discussions (FGDs). The study team conducted 2 FGDs with a total of 12 participants during the intervention development phase in 2016. In early 2017, a total of 2 additional FGDs were conducted with 5 participants after an 8-week pilot phase. Finally, in 2018, during the final 2 months of the study, 3 FGDs were conducted with 15 participants. The FGDs were transcribed, transcripts were manually coded using a deductive coding methodology to group responses by topic areas in the FGD guide, subtopics were assigned through line-by-line coding, and data were consolidated in a structured template that enabled identification of salient themes. Results from the FGDs in the formative and pilot phases informed the content and structure of the intervention and helped to identify implementation issues early in the project [47].

### Ethics Approval

Ethics approval for the study was obtained from the University of the Philippines Manila research ethics board (protocol number 2016-265-01) and the London School of Hygiene and Tropical Medicine (reference number 11631). All participants provided written consent before inclusion in the study.

## Results

### Study Population and Intervention Delivery

#### Process Evaluation Questions 1 and 2: Reach and Recruitment

Of approximately 675 patients receiving ART services at the study site during the recruitment period, 485 (71.9%) were approached by the study coordinator while attending a routine visit at the clinic, 464 (68.7%) were interested in learning about the study, and 462 (68.4%) met the eligibility criteria and consented to participate.

Reasons for refusal (21/485, 4.3%) included no need or desire for adherence support, not wanting to receive messages or calls on their mobile phone, privacy concerns, and frequent travel out of the country. Of the 0.4% (2/464) of the patients who were excluded, one was ineligible because he did not speak English and the other did not have a mobile phone.

All but 1 of the participants in the study (461/462, 99.8%) identified as male, and 98.5% (455/462) were MSM. The mean age at enrollment was 32.4 (SD 5.7) years. University or postgraduate studies had been completed by 85.9% (397/462) of the participants, and 91.3% (422/462) were employed or enrolled in university, which reflects the higher-than-average socioeconomic status of patients at the study site, a private fee-for-service clinic.

At the time of enrollment, 92.2% (426/462) of the participants were already taking ART and 7.8% (36/462) had not yet started. Of those already taking ART, perfect adherence of 100% of doses taken in the last 30 days was reported by 52.1% (222/426) of the participants, 95% to 99% adherence was reported by 26.6% (113/426), 90% to 94% adherence was reported by 12.7% (54/426), and adherence of <90% was reported by 8.7% (37/426).

Participants were followed for 48 weeks, during which time 91.1% (421/462) of the participants were retained for the study duration and active on ART at the study site, 0.6% (3/462) had withdrawn from the study but were still in care, 0.6% (3/462) had died, 3.9% (18/462) had defaulted from treatment, and 3.7% (17/462) had transferred to another clinic.

#### Process Evaluation Question 3: Fidelity

The process evaluation found that the fidelity of the intervention was low. The planned intervention consisted of daily IVRS pill reminder calls for all participants in the first 6 months of ART and weekly IVRS calls for those on ART for >6 months. During the study, only 22.1% (102/462) of the participants received the IVRS intervention, whereas 72.7% (336/462) received a scaled-down SMS text message version of the intervention. The reasons for the small proportion of participants receiving the voice calls were technology-related challenges described in the *Usability and Context* section.

#### Process Evaluation Questions 4 and 5: Dose Delivered

Of the 462 participants, 95 (20.6%) participants received a combination of voice calls and SMS text messages, 336 (72.7%) received SMS text messages only, 7 (1.5%) received voice calls only, and 24 (5.2%) received neither.

The 22.1% (102/462) of the participants who opted for IVRS services received a total of 30,940 calls during their study enrollment period (Table 1). During the calls, participants listened to 3980 health tips. Only 2 symptom or side effect reports were made. An average of 303 calls were made per participant, which included repeat reminder calls (up to 3 calls per day) if the initial call was unanswered. Of all the scheduled outgoing IVRS calls by the Connect for Life system, only 0.14% (44/31,095) of the calls failed to initiate owing to a software or platform issue.

The 93.3% (431/462) of the participants who opted for SMS text messages received 8234 messages in total: 2468 (29.97%) adherence feedback, 417 (5.06%) health tips, 2272 (27.59%) pill reminders, and 3077 (37.37%) visit reminders.

**Table 1:** IVRS^a^ and SMS text message services provided.

Services		Participants who received the service (N=462), n (%)	Total number of calls and messages delivered after enrollment (N=30,940 calls; N=8234 SMS text messages), n (%)	Number of calls and SMS text messages per participant, mean (SD)
**IVRS calls (n=102)**				
	Any	102 (22.1)	30,940 (100)	303 (324.3)
	Listened to health tip	69 (14.9)	3980 (12.86)	58 (80.1)
	Reported symptoms or side effects	2 (0.4)	2 (0.01)	1 (0)
**SMS text messages (n=431)**				
	Any	431 (93.3)	8234 (100)	19 (49)
	Adherence feedback	70 (15.2)	2468 (30)	35 (17.3)
	Health tip	11 (2.4)	417 (5.1)	38 (45.7)
	Pill reminder	10 (2.2)	2272 (27.6)	227 (187.3)
	Visit reminder	428 (92.6)	3077 (37.4)	7 (4)

^a^IVRS: interactive voice response system.

#### Process Evaluation Question 6: Dose Received

**Figure 3:**
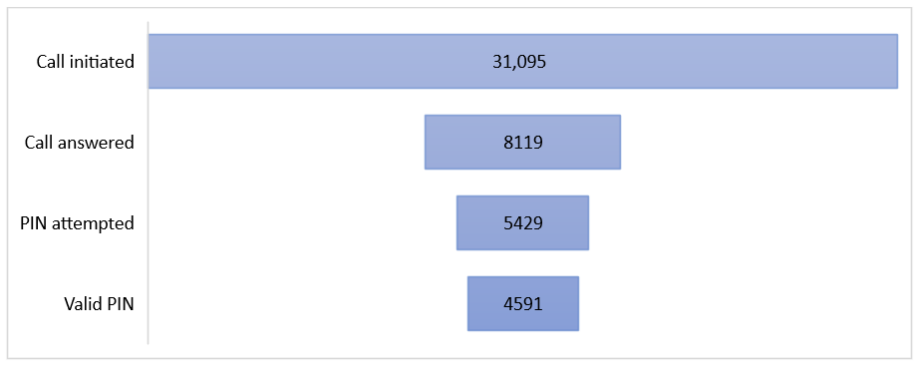
Interactive voice response system calls made and outcomes. PIN: personal identification number.

Including setup calls during the visits, of the 31,095 outgoing calls made by the Connect for Life system, 8119 (26.11%) were answered by the participants. To listen to the message, the participant had to enter their personal identification number (PIN). A PIN attempt was recorded for 66.87% (5429/8119) of the calls that were answered, and the PIN was entered successfully in 84.56% (4591/5429) of the PIN attempts (Figure 3).

Of the 2690 calls that were answered and no PIN was entered, an estimated 1846 (68.62%) went to voicemail. This estimate was based on the number of seconds the call was connected before it was automatically terminated by the software (approximately 140 seconds).

### Experiences of Participants and Providers

#### Process Evaluation Questions 7 and 8: Usability and Context

The biggest technology challenge that the project faced was frequent dial tone multifrequency (DTMF) malfunction during IVRS calls. This was reported by study participants and observed by the study staff during the process of activation of the IVRS service. During the DTMF malfunction, the system was unable to recognize the tones as users pressed number keys on their phones, resulting in invalid PINs or inability to navigate the IVRS menus. DTMF failure was suspected during an estimated 32.08% (2605/8119) of calls that were answered by participants (1767/2605, 67.83% of the answered calls where no PIN was entered and 838/2605, 32.17% calls where an invalid PIN was entered). Enrollment was temporarily suspended, and an investigation of the issue found that the DTMF malfunctions were related to the telecommunication infrastructure rather than the Connect for Life platform; therefore, it was not possible for the study team to correct the issues.

**Figure 4:**
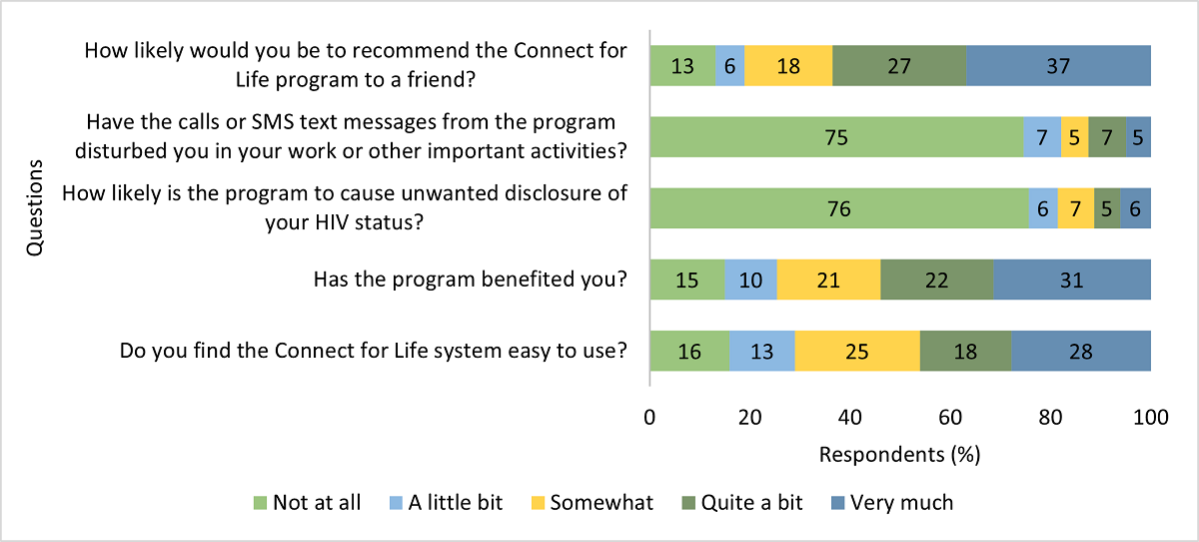
Intervention acceptability after 48 weeks (n=392 respondents).

Only 46.1% (159/345) of the participants reported that they found the Connect for Life system quite easy or very easy to use (Figure 4), indicating that ease of use can be improved.

The provider’s experience with the system was largely positive. In monthly process reports, clinicians reported that the medical record functionality facilitated easy access to laboratory results, medication history, diagnosis, and other information, which had previously been recorded in Microsoft Word documents and paper charts. Clinicians also reported that the alert function, which flagged patients with poor adherence or side effects for the clinician to follow up, was overwhelming to use. The symptom reporting alerts were useful, but these alerts were “buried” in a long list of alerts about missed doses and missed clinic visits. This occurred when participants failed to answer calls and responded to the IVRS prompts, which triggered alerts for nonadherence, resulting in high numbers of inaccurate alerts for missed doses. Clinicians recommended reviewing and updating the criteria for generating alerts.

Clinic staff also observed that across the clinic, participant compliance with attending appointments on the scheduled date and time improved from 17% before the study to >30% after the implementation of Connect for Life. They attributed this improvement to the visit reminders sent through SMS text messages. The improved on-time visit attendance saved staff time and effort by reducing the need to call patients and reschedule appointments.

#### Process Evaluation Question 9: Acceptability and Satisfaction

##### Acceptability

Acceptability questionnaires were collected at 3 time points (426/462, 92.2% completed at the 12-week visit; 335/462, 72.5% at the 24-week visit; and 392/462, 84.8% at the 48-week visit). Acceptability levels are summarized in Figure 4.

After 48 weeks in the study, 63.5% (221/348) of the participants reported that they would be quite likely or very likely to recommend the program to a friend, and 53.9% (187/347) of the participants reported that they benefited quite a bit or very much from the intervention.

Some participants reported concern over privacy and inconvenience, with 12.4% (43/347) of the participants reporting that the messages and calls disturbed them quite a bit or very much during their work or other important activities and 11.3% (39/345) of the participants stating it was quite likely or very likely that the intervention could cause unwanted disclosure of HIV status. Social harm monitoring was conducted at each study visit and no instances of disclosure were reported.

Associations between acceptability and several independent variables were explored.

##### Time on Study

There was no strong evidence of difference in the acceptability indicators at different time points after enrollment. The proportion of participants who reported that the intervention benefited them quite a bit or very much was 45.2% (128/283) at the 12-week study visit, 54.3% (188/346) at the 24-week visit, and 53.9% (187/347) at the 48-week visit (*P*=.51)

##### Time on Treatment

Among participants who had started ART <6 months before enrollment in the intervention, after 48 weeks, 65% (39/60) reported that the intervention benefited them quite a bit or very much, compared with only 51.6% (148/287) of the more experienced participants who had been on ART for >6 months at the time of enrollment (*P*=.02).

##### Frequency of Service

People who received daily or weekly pill reminders were much more likely to report that the intervention benefited them compared with those who did not receive pill reminders. This trend was consistent across all time points. At the 48-week visit, 70% (21/30) of the participants who received weekly pill reminder and 64% (9/14) of those who received daily pill reminder reported that they benefited quite a bit or very much from the intervention compared with only 51.5% (157/305) of those who received no reminders (*P*=.01).

There was no evidence of difference between those receiving daily and those receiving weekly pill reminders in terms of acceptability of the frequency of pill reminders or participants’ likelihood to recommend Connect for Life to a friend. Of those who received daily pill reminder, 14% (11/78 observations) said that there were “too many” reminders, whereas 7% (4/58 observations) of those who received weekly pill reminder said that there were “too many” reminders (*P*=.29). At week 48, a total of 80% (24/30) of the participants who received weekly pill reminders were quite likely or very likely to recommend to a friend, compared with 64% (9/14) of those who received daily pill reminders and 61.4% (188/306) of those who received no reminders (*P*=.30).

##### Other Factors

No association was observed between viral load suppression or HIV knowledge score and intervention acceptability.

##### Qualitative Feedback From FGDs and Adherence Questionnaires

Qualitative data were collected to facilitate better understanding of participants’ experiences with the system and the contextual and motivating factors influencing the use, acceptability, and usability of the intervention.

The key findings from the acceptability questionnaires and the FGDs at the end of the study were that the intervention was received positively, and participants believed that the intervention should continue after the study ended. Several main themes emerged—the importance of personalized reminders, technical challenges and usability issues, desire for health tips, and importance of social support as part of HIV care (Textbox 1).

**Main themes from focus group discussions (FGDs).**

**Personalized reminders**

- Participants liked that the intervention was highly personalizable, enabling them to select the frequency and time of calls or SMS test messages and the topics of health tips. Preferences for voice calls and SMS text messages varied. Participants also reported that they found the visit reminders and pill reminders to be helpful for their adherence; however, most patients were using their own alarms or pill boxes as adherence tools. Several participants who only received the visit reminder service expressed interest in trying the pill reminders and health tips after hearing the feedback from participants who received those components of the service:

It is an advantage being reminded at work especially when you get busy so you would not miss to take your medicine on time.

Receiving pill reminder call on a weekly basis made me more aware of the time and I think it is more beneficial to those who has tight schedule. But in my end, I never forget a dose with the aid of alarm clock.

For me, the two times [visit] reminder is fine. Actually, it is very helpful on reminding me on my next visit. There are times that I got surprised receiving the text because I already forgot that I have a follow-up visit.

**Technical challenges**

- Participants who received the calls described challenges with entering their personal identification number and with navigating the interactive voice response system (these challenges were owing to failure of the dial tone multifrequency technology) and more broadly about the hassle of responding to the prompts in the calls. Even when the call went unanswered, it still served as a prompt to take medication:

In the evening, I don’t know how to use the PIN so whenever I received the call (usually an international number) and hear the music, I already know that it is the pill reminder call. I actually can’t go through the IVR because I don’t know exactly when I need to enter the PIN... On the other hand, the call itself serves as an alarm to take my meds though I was not able to answer or enter my PIN.

**Health tips**

- Participants expressed that although they use the internet to find health information, they trusted health tips from Connect for Life more, because the information was vetted by their health care provider. They liked that the health tips included information on a range of related health topics, such as nutrition and mental health, in addition to the HIV basics. However, some participants were unwilling to receive tips via SMS text message because of concerns about privacy, and some stated that they knew someone who they could ask for health information:

In general, I think it is better that the health tips are coming from Sustained Health Initiatives of the Philippines and recommended by health care professionals. It would be more reliable as compared to information in the Google.

It’s like trivia for today, even you are on meds for a long time already.

**Social support**

- Almost all FGD participants mentioned the importance of human connection. Several participants mentioned that they would prefer to connect to a live person in addition to electronic information, especially regarding symptom management. Participants stressed the role of support from their health care providers or other patients in helping them to understand more about living with HIV:

I would like to suggest having someone to reach to answer a not so relevant question like if I have stomach-ache and I want to know if it is connected to my meds or a side effect versus to searching in Google which is sometimes inaccurate.

Exchange of experiences [is important] especially to the new patient so they would know what to do. They would feel that they are not alone, because you won’t know how to avoid feeling self-pity. At least with a support group they have someone to communicate with.

#### Process Evaluation Question 10: Cost

A description of the types of expenses involved in the implementation and the approximate costs from the Philippines setting are shown in Table 2.

**Table 2:** Costs involved in the intervention.

Aspect	Description	Cost
Cloud hosting of solution	The database and software require hosting on RDS^a^ and EC2^b^ server instance. The cost of a monthly or yearly subscription depends on the amount of storage needed and payment schedule. Our database includes data for approximately 700 patients.	US $50 per month
VOIP^c^ provider	This may be the local telecommunications company (eg, Vodacom and Globe) or a specialist service provider.	PHP 0.50 (US $0.01) per SMS text message or PHP 5 (US $0.10) per minute for voice calls
Local service provider IT support	IT support monitors the server, VOIP functionality, and software updates and manages users’ log-ins. Our local IT support provides up to 20 hours of support monthly and charges an hourly rate for additional support.	PHP 10,000 (US $200) per month
Staff	An administrative clerk, counselor, or other cadre of staff will allocate time and effort to enroll patients on the system, activate their services, monitor alerts, and update details.	Cost varies (0.1-0.5 FTE^d^ of administrator)

^a^RDS: relational database service.

^b^EC2: Elastic Compute Cloud.

^c^VOIP: voice over IP.

^d^FTE: full time equivalent.

## Discussion

### Principal Findings

During the study, >31,000 IVRS calls and 8000 SMS text messages were sent to 462 study participants. The Connect for Life system was acceptable to both participants and providers. Participants liked that the intervention was highly personalizable, enabling them to select the frequency and time of calls or SMS text messages and the topics of health tips. Feedback on the pill reminders, visit reminders, and health tips was very positive. Participants appreciated that health tips covered a variety of topics beyond HIV basics. The FGDs revealed that acceptability of the weekly adherence scores and symptom reporting functionalities of the intervention was low, as these 2 functions required lengthy navigation of the IVRS menu.

Owing to technical issues, the intervention was not implemented as originally intended, with only 22.1% (102/462) of the participants receiving the IVRS pill reminder intervention and others receiving a scaled-back SMS text message intervention. When the technical issues were first identified, enrollment in the study was paused for 3 months, while the study team assessed the cause of the issue. Ultimately, the issue of DTMF malfunction was attributed to issues in the telecommunications system that neither the telecommunications provider nor the Connect for Life developers could resolve. When enrollment was resumed, participants were provided SMS text messages rather than IVRS services. Despite the technical challenges, acceptability remained high, and only 0.6% (3/462) of the participants withdrew from the study. Following the study, the frequency of technical issues has decreased significantly, and the study site has continued to provide the service. Currently, pill reminder calls are a routine service for all new patients undergoing ART.

Notably, the technical challenges experienced in delivering the intervention were related to navigating the IVRS menu and made it difficult to distinguish whether the issues raised with ease of use or overall satisfaction were related to the technical challenges (ie, the dial tones were not recognized when keyed in) or to the product design (ie, IVRS menus were very complicated). The accuracy of the adherence scores in the weekly feedback SMS text messages was dependent on successful navigation of the IVRS process. This type of feedback may have been better delivered via a smart phone app rather than an IVRS setup. The interactive component of the IVRS system was an important aspect of the study design, which was not effectively evaluated in this study owing to the low number of participants who received this part of the service, warranting ongoing monitoring and future studies.

The scaled-back intervention provided everyone with visit reminders, which addressed part of the theory of change by improving medication accessibility through timely refills, but did little to prompt pill-taking, habit forming, and improvements in health knowledge. Individuals who received a high dose of the intervention (daily or weekly pill reminders) were more likely to recommend the intervention to others, suggesting that the planned intervention was more acceptable than the scaled-back version.

Our analysis of dose received shows that the call answer rate was low, with only 26.24% (8119/30,940) of outgoing calls answered, which is reflective of a preference for SMS text messages and chat services among the target population. The requirement of a PIN reduced exposure to the intervention, which was mostly owing to technological challenges. After experiencing technical difficulties several times, many participants stopped answering the calls. However, some mentioned that the phone ringing at the set time each day served as an effective adherence reminder.

Privacy considerations were paramount, with 11% (51/462) of the participants reporting that they had concerns about the potential for disclosure of their HIV status. Therefore, in situations where entering a PIN is a barrier to intervention exposure, practitioners can consider adapting the content to eliminate potentially sensitive health information and delivering the service with no PIN requirement.

Ultimately, the study showed the importance of choosing technologies that can function in local contexts. In low-resource settings, it may take time to scale-up technologies that will be quick to roll out in high-resource settings. Practitioners must identify service providers with appropriate capacity and ensure that patients have the skills and motivation to use the intervention. Conducting an iterative process with several pilot stages is advantageous, as it enables practitioners to identify the problems with functionality and adapt the intervention before scaling up.

An important aspect of the intervention was that, through this regular contact from the clinic, participants felt cared for and felt that their health care provider was concerned about their well-being. This social support was a key motivator for adherence. Participants requested to be able to speak to someone about side effects or for social support, suggesting that an intervention that links calls to counselors more effectively may be an area for future evaluation.

### Comparison With Previous Studies

The Connect for Life Philippines intervention was adapted from the same platform used for Call for Life Uganda and mMitra and Treatment Advice using Mobile Alerts in India. Acceptability was high in all 3 settings [11,16]. However, there were differences in the preferences and use patterns of the participants in the Philippines setting compared with those in Uganda and India. The Philippines had a high preference for SMS text messages over voice calls and a low call answer rate. The Connect for Life Philippines and Call for Life Uganda projects experienced similar challenges with network instability issues in the early stages [16].

Similar to Connect for Life and Call for Life, other mHealth interventions for people living with HIV have shown improvements in ART adherence, even where participant response rates (ie, dose received) are low [29,31,56]. For example, the PositiveLinks app used by people living with HIV in Virginia, United States, had response rates of <40% to most app prompts, but participant retention in care, CD4 results, and viral suppression improved significantly [57]. There is an important distinction between adherence to the intervention (ie, calls, app prompts, and device use) and adherence to medication.

### Strengths and Limitations

A strength of the Connect for Life platform is its scalability; the project can easily be expanded to cover a large number of sites and patients with great cost efficiency, if those facilities have access to computers and internet connectivity. To deliver the project at scale, creation of content in regional languages will be an important consideration. The platform is adaptable, as the local IT provider can add and remove new data fields and update the SMS text message content, voice files, and call flows. However, changes to the functionality of the software or interoperability with other systems will require support from the software developers at Johnson and Johnson Global Public Health. The Philippines Department of Health has plans to implement electronic reporting systems for HIV services at an aggregated level. If the department is ever to implement a patient-level electronic medical record, interoperability with Connect for Life will be an important consideration to ensure delivery at scale.

A strength of this process evaluation study is the mixed methods and participatory approach. The study used prospectively collected quantitative data on participants’ responses to the intervention and qualitative feedback from questionnaires, monthly process reports, and FGDs. The evaluation included the users of the intervention, clinical service providers, and developers of the technology platform.

The methodology addressed all key components in process evaluation for public health interventions and studies (context, reach, dose delivered, dose received, fidelity, implementation, and recruitment) [55]. Furthermore, the study included information on the technology platform, infrastructure, security, and cost, as guided by the mHealth Evidence Reporting and Assessment checklist developed by the World Health Organization mHealth Technical Evidence Review Group [35].

A limitation of our approach was that the evaluation was conducted by the same study team responsible for planning and implementing the intervention, rather than by independent evaluators. Other limitations included the convenience sampling strategy for participants in the FGDs and the low participation in the focus groups. Although the study team approached many individuals to participate, it was a challenge to identify those who were willing owing to reluctance to disclose their HIV status in a group. Furthermore, owing to transportation challenges, there was low attendance among those who confirmed their intention to participate in the groups.

Incomplete data may have affected the interpretation of the results. Of the 462 participants in the study, 440 (95.2%) attended the final study visit at week 48, and 89.1% (392/440) of them completed the questionnaire during the final visit. There may be differences in the experiences of participants who transferred out, died, withdrew from the study or were lost to follow-up, attended but did not complete the questionnaire, and completed the questionnaire.

This study focused on MSM in Metro Manila, and the study population was urban and highly educated. Participants may have had alternative adherence reminders, including self-set phone alarms and email alerts. Therefore, the results are not broadly generalizable to other contexts.

### Conclusions

mHealth interventions are useful to support adherence, as they have low replication costs and are highly adaptable to specific cultural contexts. On the basis of the findings of this process evaluation, we can guide practitioners implementing mHealth interventions to support medication adherence to consider the following recommendations:

1. The intervention should allow the participant to personalize the service based on their preferences for delivery by SMS text message or voice calls, timing of messages and calls, and selection of content.
2. Limit the complexity of the IVRS menus to reduce the “hassle” factor and likelihood of technical failures. If the navigation of menus is a key aspect of the intervention, consider using an app or a chatbot instead of, or in addition to, an IVRS system.
3. Consider how to use the mHealth intervention to facilitate human interaction. For example, certain responses to the intervention may prompt counselor-, clinician-, or peer support.
4. Ensure that the roll out of an existing mHealth technology in a new setting is an iterative process that includes robust process evaluation methods. Rigorous pilot-testing is needed to ensure technical function. Work plans should include ample time and budget for adaptation of the technology.

The Connect for Life mHealth intervention to support adherence to ART had high participant satisfaction and acceptability. However, the feasibility of the intervention was dependent on the reliability of local telecommunications networks, and poor reliability of the local mobile networks had a large impact on the intervention’s usability, fidelity, and dose received.

The process evaluation allowed us to better understand the preferences and use patterns of mHealth services by MSM in the Philippines. This will enable the effective scale-up of mHealth services for this key population, which is essential in the context of the dual HIV and COVID-19 pandemics, where more services must be delivered virtually.

## Appendix

Multimedia Appendix 1 Process evaluation methodology.

## Abbreviations

ART: antiretroviral therapy
DTMF: dial tone multifrequency
FGD: focus group discussion
IVRS: interactive voice response system
mHealth: mobile health
MOTECH: Mobile Technology for Community Health
MSM: men who have sex with men
PIN: personal identification number
SHIP: Sustained Health Initiatives of the Philippines
