# Supplementary material for: Delivering an mHealth Adherence Support Intervention for Patients With HIV: Mixed Methods Process Evaluation of the Philippines Connect for Life Study
Source: JMIR Form Res. 2022 Aug 12;6(8):e37163. doi: 10.2196/37163 (PMC9419042; doi:10.2196/37163)
Supplement: Multimedia Appendix 1 [file formative_v6i8e37163_app1.docx]

[Multimedia Appendix 1: Process Evaluation Methodology]

| **Component** | **Process evaluation question** | **Data sources** | **Tools/procedures** | **Data analysis or synthesis** | **Reporting** |
| --- | --- | --- | --- | --- | --- |
| *Reach/recruitment* | 1. What proportion of participants receiving services in the study site opted into the Connect for Life intervention? 2. What procedures were followed to recruit participants into the intervention? | Screening records | Review screening records to determine the screening and enrollment rates  Recruitment script and SOP^a^ | Calculate the proportion of individuals in the study site who were screened for inclusion in the study/intervention, the proportion of those who screened who were eligible, and the proportion of those eligible who enrolled in the study. Characterize main reasons for refusal. | Formative–Examined during the study enrollment period to identify intervention coverage of clinic population and direct recruitment efforts.  Summative–Summarize after study enrollment period concluded. |
| *Fidelity* | 1. To what extent was the Connect for Life intervention implemented as planned? | Connect for Life system reports | Review system generated reports (call/SMS text message logs) to determine which intervention functionalities were activated for each participant and monitor changes. | Calculate the proportion of participants who received the intended intervention (according to the proposed dose in the recommended configuration of services). | Formative–Regular informal feedback to study coordinators and clinicians.  Summative–Summarize overall intervention fidelity after all study visits completed. |
| *Dose delivered* | 1. How many daily and weekly pill reminders, health tips, clinic visit reminders were generated by the Connect for Life System? 2. What proportion of Connect for Life calls were delivered successfully and what proportion had technical failures? | Connect for Life system reports | Review system generated reports (call/SMS text message logs) to determine the total number of calls and SMS text messages delivered. | Calculate the total number of SMS text messages sent (per category), calls generated from the Connect for Life system, and proportion of calls with technical failures. | Formative–Regular review of system reports by IT^b^ support and by study staff to monitor system functionality.  Summative–Summarized after all study visits completed. |
| *Dose received* | 1. What was the level of interaction between the participants and the Connect for Life system prompts? | Connect for Life system reports | Review system generated reports (call/SMS test message logs) to determine the proportion of various call outcomes and describe participant responses to prompts in the interactive voice response system (IVRS). | Calculate the proportion of calls answered, average length of calls answered, ratio of calls to adherence reports, levels of reported adherence, and number of health tips heard, and symptom reports recorded. | Formative–Monitor technical failures.  Summative–Summarize after all study visits completed. |
| *Usability/context* | 1. Does the Connect for Life mHealth^c^ solution function as intended? 2. What are the barriers to and facilitators of implementing the Connect for Life intervention in the local context? | Clinic staff and participants | Observations of study staff and feedback from participants.  Focus groups conducted at the design stage, after 8 weeks of pilot-testing, and at the end of the study. | Narrative description of findings regarding usability of the Connect for Life intervention  Qualitative analysis of focus group discussion transcripts. | Formative–During the initial pilot phase of the intervention, to identify and resolve intervention delivery challenges.  Summative–Describe the experience of study staff and participants who used the Connect for Life platform; the strengths and limitations of the solution; and contextual factors that influenced the fidelity, usability, acceptability, and cost of the intervention. |
| *Acceptability/*  *satisfaction* | 1. What do participants like/dislike about the intervention and what is their opinion of its effectiveness? | Participants | Focus groups conducted at the design stage, after 8 weeks of pilot-testing, and at the end of the study.  In-person questionnaires, self-administered at 12-, 24-, and 48-week study visits. | Qualitative analysis of focus group discussion transcripts.  Descriptive analysis of Likert scale scores regarding intervention acceptability and participant satisfaction and inferential analysis of scores with participant characteristics as independent variables. | Formative–Incorporate findings from focus groups conducted during the intervention design phase into the intervention content and delivery plans.  Summative–Summarize findings from quantitative analysis of questionnaires and qualitative analysis of focus group discussions after the conclusion of the study. |
| *Cost* | 1. What is the cost to implement the Connect for Life intervention in the local context? | Administrative records | Review of administrative records to extract rates for infrastructure, telecommunications, and staffing of intervention. | Determine the baseline costs to set up the Connect for Life System and the average per participant cost to deliver the intervention. | Summative–Summarize after all study visits concluded, plan for the sustainability of the project beyond the study period. |

^a^SOP: standard operating procedure.

^b^IT: Information technology.

^c^mHealth: mobile health.
